# Physical Activity and Mobile Phone Apps in the Preschool Age: Perceptions of Teachers and Parents

**DOI:** 10.2196/12512

**Published:** 2019-04-17

**Authors:** Anna Ek, Johanna Sandborg, Christine Delisle Nyström, Anna-Karin Lindqvist, Stina Rutberg, Marie Löf

**Affiliations:** 1 Department of Biosciences and Nutrition The Innovative Use of Mobile Phones to Promote Physical Activity and Nutrition Across the Lifespan Research Group Karolinska Institutet Huddinge Sweden; 2 Division of Pediatrics Department of Clinical Science, Intervention and Technology Karolinska Institutet Huddinge Sweden; 3 Division of Community Medicine Department of Medical and Health Sciences Linköping University Linköping Sweden; 4 Division of Health and Rehabilitation Department of Health Sciences Luleå University of Technology Luleå Sweden

**Keywords:** child, preschool, mHealth, physical activity, parents, school teachers, qualitative research

## Abstract

**Background:**

Physical activity (PA) is already beneficial at the preschool age. In many countries, young children spend most of their days in the preschool setting, making it a common arena for PA interventions. Mobile health tools are becoming increasingly popular to promote PA in different populations; however, little is known about the interest for and how the preschool setting could incorporate such a tool.

**Objective:**

This study aimed to examine how teachers and parents perceive PA in preschool-aged children in general and their perceptions of how a mobile phone app could be used to promote PA in the preschool setting.

**Methods:**

Semistructured interviews were conducted with 15 teachers (93%, [14/15] women, mean age 43.5 years, 47%, [7/15] with a university degree and 10 parents [91%, 9/10] women, mean age 38.9 years, all with a university degree) recruited from 2 urban preschools in central Sweden. The interviews were recorded, fully transcribed, coded, and analyzed using thematic analysis by means of an inductive approach.

**Results:**

The analysis revealed 4 themes: (1) *children are physically active by nature*, (2) *the environment as a facilitator or a barrier*, (3) *prerequisites of the adult world*, and (4) *an app in the preschool setting—challenges and possibilities*. Parents and teachers perceived preschoolers as being spontaneously physically active; however, high-intensity PA was perceived as low. The PA was specifically performed during the day in the preschool. Identified facilitators of PA were access to safe and engaging outdoor environments such as forests, spacious indoor areas, and adult involvement. Adult involvement was considered especially important for children preferring sedentary activities. Identified barriers for PA were restricted indoor and outdoor space, rules for indoor activities, and lack of adult involvement because of time constraints. The teachers perceived that they had limited skills and experiences using apps in general, although they also acknowledged the increasing role of technological tools in the curriculum. Thus, the teachers expressed an interest for an app designed as a support tool for them, especially for situations when PA was limited because of perceived barriers. They suggested the app to include accessible information regarding the health benefits of PA in children linked to a library of activities for different settings and seasons. Parents suggested interactive app features including problem-solving tasks and music and dance, but not video clips as they made children passive.

**Conclusions:**

Vigorous PA was perceived as low in preschool-aged children. Future tailoring of interventions in the preschool setting should work around barriers and support facilitators to PA, especially PA of high intensity. In such work, an app could serve as a source of inspiration for PA in different ages, settings, and seasons and thus reduce environmental and structural inequalities in the preschool setting.

## Introduction

### Physical Activity Levels in Preschoolers

Higher physical activity (PA) levels are associated with improved metabolic and psychosocial health as well as motor and cognitive development in preschoolers [1]. In addition, preschool-aged children who spend more time in vigorous PA have a more favorable body composition and better physical fitness (ie, cardiorespiratory fitness, motor fitness, and muscular strength) [2]. Establishing a habit of being physically active during childhood increases the likelihood of being physically active as an adult [3,4]. Globally, data show that many children aged 5 to 17 years do not meet the recommended levels of 60 min of moderate-to-vigorous PA (MVPA) per day [5], and Sweden is no exception [6]. Few studies have investigated the fulfillment of PA in the newly developed 24-hour movement guidelines for the early years, that is, 180 min of total PA, of which at least 60 min is MVPA [7-9]. However, previous Swedish data have shown that only one-third of 4-year-old children reached 60 min per day of MVPA [10]. Correspondingly, another recently published Swedish study showed that children only spent 7 and 12 min per day on average on vigorous PA at 4 and 5 years of age, respectively [2]. Considering the pronounced negative health effects of physical inactivity, actions to improve children’s PA levels are required, especially activities of higher intensity.

Traditionally, PA interventions targeting preschoolers use the preschool environment [11,12]. The design of such interventions has primarily been face-to-face education combined with written information and interactive pedagogic materials [13]. This intervention design has proven well accepted, although results vary [14,15]. The advantage of interventions in the preschool setting is that it reaches all children. In Sweden, 84% of all 1- to 5-year-old children spend a significant part of their day at preschool [16]. Furthermore, and contrary to many other settings for interventions, preschools also reach children from different sociodemographic backgrounds. For example, approximately 78% of all 1- to 5-year-old children with a migrant background attend preschool [16]. However, traditional interventions are often difficult to scale-up as face-to-face education require extensive resources, and thus, alternative delivery options should be considered.

### Mobile Health to Promote Physical Activity in Preschoolers

In the past decade, interest in the use of mobile phones for delivering lifestyle interventions has increased [17-21]. In comparison with traditional lifestyle interventions, mobile health (mHealth) programs are more flexible and can be tailored to meet individual needs [17]. Moreover, mHealth interventions have the advantages that they can be delivered anywhere and at any time and they can easily be scaled-up [17,18]. However, few mHealth interventions exist for promoting PA in the preschool age group, and so far, they have only targeted parents [22]. In Australia, an intervention aimed to decrease screen time in preschool children aged 2 to 4 years by sending supportive text messages (short message service, SMS) to parents [23]. The study design proved effective with increased PA in the children and was appreciated by the parents [23]. Similarly, another intervention included delivering text messages to parents to support healthy habits as an intervention for children with obesity [24]. The intervention was well accepted, although no effect was seen for child weight status or behavior [24]. In Sweden, the Mobile-based intervention intended to stop obesity in preschoolers (MINISTOP) study used a mobile phone app to promote healthy eating and PA in 4.5-year-old children [25]. Although no significant difference in weight status was found between the intervention and control group, the intervention group improved their composite score based on fruit and vegetable consumption, PA, and fat mass index. Interestingly, this improvement was higher among children with a higher fat mass index [25]. Importantly, all the aforementioned mHealth interventions were well accepted and used by the parents. Taken together, the results from these interventions suggest that an app targeting parents can be used to influence lifestyle behaviors in young children. However, such apps may also target preschool teachers who spend many hours with the children, and tablets are already being used for pedagogical purposes within the Swedish preschool curriculum.

To develop an app for the preschool setting, we need to know more about the interest in such a tool as well as appropriate content and features to include and to whom the app should be designed for. Thus, a first step is to collect such information from potential end users, that is, teachers and parents. Including the views of end users can improve the intervention’s compatibility and increase the likelihood that it will be effective and sustained [26-28]. Furthermore, information regarding perceptions of PA in general needs to be collected to ensure the app reflects the users’ expectations. For example, this could include the following: what are the types of PA that is conducted currently, what activities are preferred by children and teachers, what role do adults have in promoting PA in preschoolers, and what are the facilitators and barriers of PA. Therefore, the aims of this study were to explore how teachers and parents perceive PA in preschool-aged children and how a mobile phone app may be used to promote PA in the preschool setting.

## Methods

### Recruitment

A total of 2 preschools representing 2 cities (Norrköping and Linköping) in central Sweden were asked to participate in this study. The preschools were chosen based on previously established contact between the research group and the head of the preschool. The preschools differed in size (48 children in Linköping and 30 children in Norrköping). In Linköping, most of the parents were born in Sweden; however, in Norrköping, one-third of the children had parents born outside of Sweden. All staff at the 2 preschools were informed about the aims of this study and were invited to participate in semistructured interviews. To recruit parents, posters with information about the study were put up on bulletin boards at the 2 preschools so that interested parents could contact the research team. The teachers’ interviews were coordinated between the research staff and the head of the preschool. The parents emailed the researchers so that interviews could be scheduled at an appropriate time. The interviews were conducted between January and April 2017. The study was approved by the regional ethical board in Stockholm (October 10, 2016, dnr: 201612099-32). Informed written consent was obtained from both the parents and teachers participating in the semistructured interviews. The reporting of this study follows the Consolidated criteria for reporting qualitative research checklist; see Multimedia Appendix 1 [29].

### Semistructured Interviews

The teachers and parents were interviewed individually in a separate room at the preschool where they worked or had their child enrolled. The interviews were conducted in Swedish face-to-face by the female researchers AE and JS and lasted for 12 to 42 min. For educational purposes, AE, postdoctoral fellow and registered dietician with previous experience in performing semistructured interviews with adults regarding children’s lifestyle behaviors, conducted the interviews in Norrköping, whereas JS, nutritionist and master student, observed. JS conducted the interviews in Linköping. All participants were asked the same set of main questions (see Multimedia Appendix 2), with follow-up questions tailored to individual responses following a semistructured design. The main questions were developed and discussed in our research group, which has extensive experience with PA interventions and mHealth for preschoolers. The interviews were recorded (audio) and then fully transcribed by JS.

Before the interviews started, the interviewees were given a short introduction about the aim of the study and the research group. Teachers and parents answered questions regarding their age, sex, country of birth, and education level. In addition, parents gave information about how many children they had and the children’s ages.

### Data Analysis

To analyze the interviews, thematic analysis was conducted, which is a commonly used method to describe qualitative data [30]. Thematic analysis is a flexible and useful tool to clearly summarize key features and provide a description of the dataset [30]. The transcribed material was read and reread in an active way by AE and JS and then separately coded using an inductive approach (ie, data-driven; without trying to fit data into a pre-existing coding frame or the preconceptions of the researcher). When all of the data had been coded, the process of finding themes was initiated. The codes and the interpretation of the data were discussed between the 2 coders over the phone and during face-to-face meetings as well as in email correspondence. Disagreements between the coders were resolved in discussions before the codes were sorted into potential themes. The themes were identified at a semantic level (ie, it is what the participants explicitly said that is being analyzed and not the potentially underlying meaning) [30].

Descriptive data for sociodemographic variables (means, SDs, and percentages) were analyzed using IBM SPSS statistics version 23 (IBM, Armonk, NY, USA).

## Results

### Participants

All but 1 (because of illness) of the consulted teachers agreed to participate in the interviews (N=15). The majority of the teachers were women (93%, 14/15) and born in Sweden (93%, 14/15). Almost half of the teachers had a university degree (47%, 7/15), and the other half had up to a 3-year secondary school degree (53%, 8/15).  The mean age of the teachers was 43.5 years (SD 13.9; min-max 20-65 years). All of the participating parents were born in Sweden (N=11), the majority were women (10/11, 91%), and all had a university degree. The mean age of the parents was 39.8 years (SD 5.2; min-max 32-47 years). The parents had, on average, 2.4 children (min-max 1-5) between the ages of 1 and 15 years. The mean age of the children attending preschool (n=15) was 3.7 years (min-max 2-5 years).

### Themes From the Thematic Analysis

**Figure 1:**
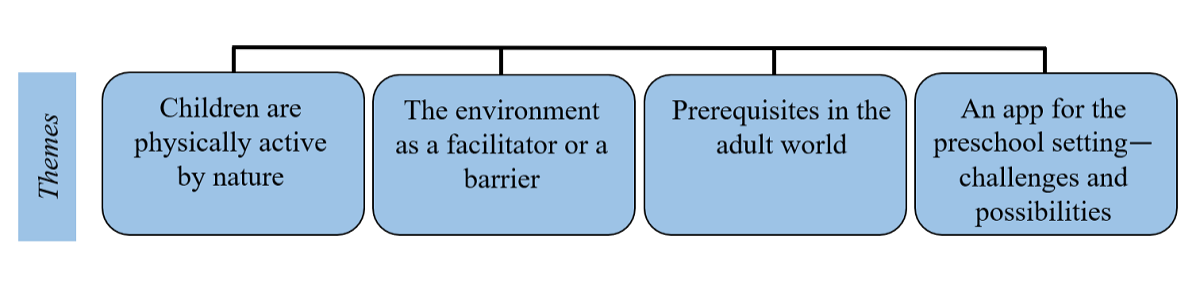
The themes identified in the thematic analysis.

A total of 4 themes were identified in the thematic analysis (see Figure 1). Each quote has been labeled with the following: P for parent or T for teacher, the participant number, and M for male and F for female.

#### Children are Physically Active by Nature

The preschool was considered by both teachers and parents as the most important setting for children’s PA because of the long hours spent there. Teachers and parents also expressed how preschoolers are spontaneously physically active and move around all the time. However, they also indicated that there was room for more high-intensity PA as it was perceived as low by both teachers and parents. There was a consensus about the need of everyday PA for children’s overall physical and psychosocial health and development. Among the positive effects of PA, teachers found active play, preferably outdoors, fundamental for children to be able to concentrate on less active indoor activities during the day. After active play, the environment often became more harmonious with less conflicts and more responsive children. The effect on the children’s mood was also experienced by parents who expressed that there were less conflicts and overall better family dynamics when they had spent time outdoors during weekends:

On the weekends we always try to spend time outdoors. We have noticed such a big difference in all our children that the days when we get outside in the morning, they are so much better...They (the children) are happier, have more energy, have plenty of ideas of what we can do, are more creative and want to play. You know, more balanced in every way, easier to deal with. If we don’t, there is more nagging, fuss and arguments between them and they don’t have the energy, and they don’t really want to...

P8F

Teachers and parents also agreed that children need a variety of PA, both spontaneous and more structured and planned activities. However, the characteristics of the child often decided what type and intensity of PA were preferred, as all *children are different*. Teachers described how the need for physical challenges increased as the children grow older. In addition, teachers suggested active transportation (eg, walking as transportation) as a natural way to increase PA. However, they perceived that today’s children were not used to active transportation and that the children found walking both difficult and exhausting. A contributing factor for this perceived change over the decades was suggested to be that parents often drive the children by car or push them in the stroller. Thus, the majority of the teachers thought that children need to practice walking and also be challenged in uneven and hilly environments to strengthen their muscles. This was in some ways supported by the parents, and the lack of active transportation was often explained by a lack of time or practical limitations:

I believe children need to be active in hilly terrain to strengthen their muscles. I have noticed that the children have poor muscle strength, they get exhausted and tired right away when you are out for a walk...

T15F

#### The Environment as a Facilitator or a Barrier

According to teachers and parents, PA was typically associated with factors in the outdoor environment such as a spacious yard, hills, open areas (eg, a soccer field), trees to climb on, and paved areas to ride tricycles on. Under these conditions, the teachers expressed that they could engage children in PA all year round; however, in the winter, many layers of clothing could sometimes limit movements for the youngest children. Contrary to the facilitators for PA, a small flat yard with no open space was perceived to have a negative impact with regard to children engaging in PA, which was the situation for 1 of the preschools. Thus, the outdoor environment and the location of the preschool were brought up as important factors when planning for an optimal preschool environment. For example, the forest was mentioned as the perfect place for diverse activities by both teachers and parents and a popular place to visit. Thus, the location of the preschool decided if visits were possible on a regular basis. In addition, parents expressed that a backyard or other safe outdoor areas and playgrounds as well as living in a larger home offered opportunities for the children to play more freely and therefore be more physically active:

I wish we had a more encouraging yard...more opportunities for climbing and doing balance exercises...a hill where the children could run up and down for instance, now it’s just a small flat yard...

T3F

Indoors, teachers described children to be less active; however, this variation in PA was described as important for the development of fine motor skills (eg, drawing and putting together jigsaw puzzles). Teachers and parents perceived limited indoor space as the most important barrier to PA, as the activities often became disturbing, noisy, and even unsafe. To maintain a pleasant and safe indoor environment, calmer types of activities were encouraged, and the children were often divided by teachers into smaller groups engaging in different types of activities. In the preschool situated in the larger facility, the teachers described that the children could be taken to different rooms to perform different activities. For example, to the music room or a larger room where the children could engage in active play. In the other preschool, teachers found it more difficult to encourage indoor PA because of the limited space. Teachers explained that with larger groups of children, they more often encouraged calm activities:

It easily gets messy when there are many children indoors and it gets noisy in a different way, sonically, and we don’t really have access to larger facilities that are often required.

T16F

#### Prerequisites in the Adult World

The teachers described that the daily operations in the preschool were governed by several factors: the preschool curriculum and learning goals, the applied pedagogical approach, time constraints, the possibility to get temporary staff at times of illness, and the number of children. Low staff density made it difficult for teachers to attend to the PA needs for all children. For instance, trips to the forest or other planned activities could be canceled on these occasions. A further consequence of low staff density was the lack of time and opportunity to plan and come up with new engaging activities for the children.

The 2 preschools investigated had different pedagogical approaches: 1 was more permissive when it came to PA indoors, whereas the other one had stricter rules of how the children could be active indoors. The latter also had a clear schedule for mornings devoted to calmer indoor activities (ie, Montessori shift) and afternoons spent outdoors (ie, free play). The teachers explained that there were many goals in the preschool curriculum that they were obligated to fulfill, which could also stand in the way for PA. Thus, a way to include PA with a pedagogical approach was for teachers to use rhymes and fairy tales that encouraged the children to be physically active. The guidelines and policies from higher authorities impacted the focus in the preschool, making it easier to implement something if it had precedence from a higher authority. However, a teacher clarified this statement by saying that even though there were many goals to fulfill, the means of how the preschool chose to do it was up to the teachers, thus leaving them with opportunities:

I love to go with the children to the forest. It doesn’t happen that frequently now since I work with the youngest children and because there are so many children in the group. Therefore, we don’t get to go so often, but I really enjoy going to the forest. In the forest, you discover things together. With the older children there is more free play, we build forts and you can see their imagination take over and they climb. I also think it is fun to be in the tobogganing hill, the activity there feels like good quality and you can see the children run up and down. They exhaust themselves a bit and I think that’s good.

T16F

...to see the joy in the children’s eyes when they (realize) “I can, I could climb up” or “I can run this far” that is also really, really fun...

T5F

The long hours at the preschool made parents and teachers agree on the important role of the teacher to facilitate PA in preschoolers. Teachers described how their own interest, attitude, and awareness regarding PA affected both the duration and types of activities offered in the preschool:

I really enjoy going tobogganing with them (the children), and I think it’s really fun to bicycle together because then we (the teachers) also go bicycling on the small bikes and you get this close contact with the children...I might not be a big fan of soccer because I’m not good at it, but it’s a lot of fun when we play music outdoors and do gymnastics together out in the yard, that’s really fun.

T10F

Although PA was part of the curriculum, teachers described how it was their individual characteristics that decided to what extent PA was used as a means to meet other curriculum goals. To make PA less dependent on individual characteristics of the staff, they highlighted the importance of planning PA together in the staff group and prioritizing PA in the weekly schedule. Several teachers expressed how it was their responsibility to acknowledge the various needs and characteristics of the children, for example, to encourage sedentary children to engage in more active play and to challenge children who are already physically active:

I think most of it (PA) depends on the adults, what attitude you as an adult have to a certain activity. If you yourself think that it is fun, then you make sure that the children get involved. Because there are opportunities everywhere even if you don’t have a gym and those kind of things. There are opportunities, if you have imagination, and then it depends on the adults.

T11F

Both teachers and parents described how children find activities more fun when adults participate and how, for some children, adult initiatives were crucial to getting them involved in active play. Parents created opportunities for PA by being outdoors with their children and buying special equipment. Signing their child up for organized PA was another support parents offered, such as gymnastics or dance classes. Contrary to this, parents also described themselves as the biggest barrier to their child’s PA. Parents’ busy work schedules and children spending long hours at the preschool left little or no time, nor energy, for PA during weekdays. The ambition of being outdoors during the weekends was also a challenge as other things tended to get in the way. Similar to the preschool, house rules could also limit the children’s ability to be active as parents often found it noisy and disturbing:

It is more often that we as parents limit the children; when they want to start moving and play–the kind of games that we often, as adults, limit by saying “no, indoors we walk”, we limit them in their play because it takes up too much space or gets too loud and noisy, even though they need it daily.

P20F

#### An App in the Preschool Setting—Challenges and Possibilities

All teachers except 1 stated that they had not yet started working with tablets in their organization. Most of the teachers expressed that they felt uncertain and not comfortable using tablets and apps. The variation in technical skills among the staff affected the implementation of the use of tablets. Teachers explained that it would also take time for the children to get used to the tablets as a component in the preschool and to learn how to use it as intended in the curriculum:

I find it a bit difficult to see (the need)...I struggle to find the role for Ipads in the preschool setting...

T14F

Even though some could not see the need for an app in this age group, others did come up with ideas on how an app could contribute to the promotion of PA in the preschool setting. Suggested content for the app was information regarding the positive benefits of PA for children, as this was regarded as a motivating factor for teachers to use the app. Practical tips on active play including music and dance and how to create a stimulating environment for PA were also desirable attributes to include in an app. The teachers also stressed that it was necessary that the app could be used in several different settings: indoors, outdoors, in the forest, in the yard, different seasons, for different ages, and various numbers of children:

...an app with features for when you are in the forest or when you are in the yard or in a large room to play...or if you have a small room, what you could do there...that would be very interesting...

T14F

The parents were positive toward the use of an app in the preschool setting. Furthermore, the parents identified role models such as a popular personality from a children’s TV program or other children in the same age group as important elements within an app to engage the children. Some parents also described interactive games involving treasure hunts and reward systems (eg, Pokémon Go) as potential app features:

Gathering things to go on treasure hunts are fun and some kind of positive reinforcement like a point system, also, Pokémon Go has a nice design and fun figures–my five-year-old likes it.

P21F

Another function that was suggested was the use of videos as a way of demonstrating PA and various exercises. However, video clips in the app revealed some contradiction, as screen time, in general, was a concern for the effect it could cause on the child’s imagination, creativity, and own initiatives for other activities. One parent stressed that video clips included in an app promoting PA should not be too captivating as this could instead turn the child into an observer, whereas a teacher expressed that videos can be a source of inspiration:

If we put on music and just dance together then we have a really good time, it’s a spontaneous activity, but as soon as a video comes on they stop. Even though there’s music they become passive observers.

P8F

An app that can inspire through videos of different activities and in the preschool both how you can do it indoors and outdoors and pictures and maybe also that these videos are with other children so that the children here can relate to themselves and to the preschool setting.

T19M

The idea of who should use an app differed between the teachers. Some thought that it should only be used as a tool by the teachers, whereas others thought it should be used by both the teachers and the children. However, all teachers agreed that the children should be involved in some way and that the use of tablets and apps should have a pedagogical value and not be used the same way as it is in the home environment (ie, mostly sedentary).

## Discussion

### Principal Findings

In this study, we interviewed parents and teachers regarding their perspectives of PA in preschoolers. The responses regarding PA were then followed with questions about what role a mobile phone app could have to support PA in the preschool setting and what features such an app should include. The interviews revealed 4 themes: *children are physically active by nature, the environment as a facilitator or a barrier, prerequisites of the adult world, and an app in the preschool setting—challenges and possibilities.* We found an understanding for the important role of PA for optimal child development in our sample. The long hours children spend in the preschool highlighted the important role of the preschool teachers for sufficient PA. However, both parents and teachers found preschoolers to engage in little vigorous PA and identified environmental and structural facilitators and barriers for PA. To overcome the proposed barriers, creative solutions for how to support preschoolers’ PA were welcomed. A mobile phone app promoting PA in the preschool setting was seen as a viable option to support teachers.

### Facilitators and Barriers to Preschoolers’ Physical Activity

Our results confirm and extend the results from a recently published review by Hesketh et al [28] on qualitative research regarding parents’ and care providers’ perceptions of facilitators and barriers on preschoolers’ PA [28]. First, in our study, the preschool was perceived to be the most important setting for children’s PA because of the long hours spent there, which is in line with previous research [28,31]. This is especially important to recognize when performing interventions in countries where children spend most of their days at preschool, such as in Sweden. Furthermore, PA in this young age group was generally regarded as something that occurred naturally and spontaneously and that children engage in PA if opportunities are provided. However, both teachers and parents agreed that higher-intensity PA and more diverse and challenging PA (eg, climbing and balancing) could be offered to children more often. The reason for it not being offered was often because of environmental and structural barriers (eg, small indoor and outdoor facilities, low staff density, and large groups of children) and time constraints (eg, little time to plan for PA). Contrary to our findings, in the review by Hesketh et al [28], the weather (eg, children being affected by too hot or too cold weather or children getting dirty outdoors) and safety (eg, when climbing) were also recognized as barriers to vigorous and challenging PA [28]. All these barriers need recognition, as reports from different countries show that the level of PA in preschools is low and the levels of sedentary behaviors are high [8,10,32] and interventions are warranted. The solutions on how to promote high-intensity PA may actually not be very difficult to find. Positive findings from the mHealth trial, MINISTOP, have shown that relatively small changes in the intensity of PA can lead to improvements in preschoolers’ body composition and physical fitness [2]. More specifically, this was observed when 5 min per day of sedentary behavior and low PA were substituted for 5 min of vigorous PA [2]. Increasing vigorous PA by at least 5 min per day could easily be implemented in preschools given the long hours the children spend there. Thus, an app intervention including a short daily exercise program has the potential to help in making this change. In addition, this structured form of an aerobic program tailored for young children was appreciated by the teachers in our sample. Furthermore, such a program would also support teachers as role models for PA and to succeed in what the teachers in our sample perceived as their responsibility, to ensure sufficient daily PA for all preschoolers to complement the low levels of PA observed in the home environment [31,33-35].

Our findings also showed how the teachers may become a barrier to PA, especially in the indoor setting where active play easily became loud and rowdy, which increased the risk of children getting hurt. However, if the facilities had more space or had an indoor playroom where the children were allowed to run around, teachers acknowledged how they would like to encourage active play more indoors, especially during winter. The preschool’s structures (eg, the pedagogical approach, ie, stricter rules for PA indoors) and the comprehensive preschool curriculum (ie, many goals to fulfill) also made the teachers to act as a barrier for PA. The ambiguous role of teachers was also recognized by Hesketh et al [28]. Training of staff has been suggested to facilitate PA in the preschool setting and even out the impact of individual characteristics among the teachers [15,36-38]. Although staff training was mentioned in our sample, the participating teachers especially requested more time for planning activities as well as an increased staff density as ways to promote PA. It is possible that an app could be a support in situations when time and space are limited. Previous research found that text messages were appreciated by parents and were effective in making preschoolers more active [23,24]. Therefore, a text message function with suggestions for daily activities could also be an option in the preschool setting to counteract the environmental and structural barriers.

Teachers found it especially challenging to support the older preschoolers’ need for more diverse and challenging PA that requires a larger and more challenging yard (eg, hills and open spaces with trees) or regular trips to parks, fields, and the forest. The difficulties in keeping older preschoolers active were recognized in a previous study showing that preschoolers’ PA decreases with every yearly increase in age [33]. The facilitators to PA found in this study are consistent with the existing literature, suggesting that preschools with more supportive environments (eg, portable and fixed play equipment, staff education and training, positive engagement by teachers in the playground, less children per square meter in the yard, and the presence of vegetation and open play areas) increase vigorous and overall PA levels in children and decrease sedentary behaviors to a greater extent compared with preschools with less supportive environments [15,36-38]. If the environmental restrictions bring on a decrease in PA already in this young age, a first step for interventions would be to find accessible creative solutions, preferably low cost, to support factors of greatest importance to PA as perceived by the target population [28]. This was also what our sample recognized as a role for an app in the preschool setting.

### An App Promoting Physical Activity in the Preschool Setting

To our knowledge, this is the first study to investigate if and how an app could be used to increase PA in the preschool setting and what specific features to include; thus, no comparable data exist. Below, we summarize suggestions based on our findings for specific app features and highlight some of the possibilities and challenges with regard to implementing and using apps in the preschool setting. Our results show that the idea and use of any apps in the preschool setting were still new to most of the teachers, even though the current Swedish preschool curriculum includes the usage of technological tools [39]. Given the diverse technical skills and lack of time for planning in the preschool, apps targeting the preschool environment need to be pedagogical and user-friendly to maximize usage. Hence, when developing an app for this setting, it is particularly important that the formative work is conducted through close collaboration between teachers and app developers at an early stage to ensure that the specific user requirements are fulfilled. In addition, when developing an app for use in the preschool environment, we need to be cautious so that we do not introduce screen-related issues such as conflicts between children and between children and teachers, reduce the children’s own creativity for other activities, and features that could make children inactive (eg, watching video clips). For these reasons, the teachers suggested that the app should primarily be aimed at them. An app would benefit from including accessible information about the health benefits of PA in children linked to a library of activities promoting child development in different ages, for example, activities supporting motor skills and cognitive development such as different types of music and dances with diverse coordination practices. As discussed above, the suggested activities would preferably be independent of environmental and structural barriers to PA. For these features, the teachers should be used as a source of knowledge and creativity as rhymes and interactive storytelling are elements they often use in their daily work. Features that could be directed to the children to get them moving and interacting at the same time were suggested; however, this depends on the child’s age. An example of such an app with such features is Pokémon Go [40]. In a qualitative evaluation of what made children appreciate the Pokémon Go app, the strongest and most prevalent answer was the collaboration with others [41]. Thus, gamification elements might be interesting to incorporate into an app. Finally, it is relevant to note that costs for the tablets were not identified as a barrier in our study. Most likely because Sweden, as a very high-income country, already uses technical devices as part of the curriculum; however, the high cost of these devices may be an issue in other countries.

A theoretical framework for guidance in the development of an app was not acknowledged by the parents and teachers in our sample; however, this is recommended for interventions [42]. The social learning and social cognitive theory are often used in interventions targeting PA and children [43-45]. These theories would support the features suggested in the interviews, for example, teachers as role models and children interacting in a dance or a game and being rewarded and getting feedback for achievements in interactive games. In summary, an app promoting PA in the preschool setting is warranted, especially as an easy and ready-to-use support tool for teachers to find solutions for the children to be active when the environment is not ideal.

### Strengths and Limitations

A strength of this study is the qualitative approach, which is a tool for developing feasible and sustainable interventions in different populations [46]. Furthermore, the semistructured interviews enabled a more detailed and rich description of the participants’ perceptions compared with questionnaires that leave participants with little room to elaborate on their opinions. In addition, the wide age span of teachers allowed us to capture perspectives from those with various amounts of work experience and views and use of apps. Limitations are the convenience sample of 2 preschools: both were situated in affluent areas and participating teachers and parents were well educated. It is possible that this distribution in education had an impact on the results, and it would have been valuable to broaden the interviews to a more diverse sample. The homogeneity of the parents might have been because of the way they were recruited, as they approached us after reading about the study on the preschool’s bulletin board rather than all parents receiving a personal request. Although our sample was homogenous, we obtained a saturation of responses within this group. Furthermore, the factors presented do represent what other similar studies have found in more heterogeneous populations [28]; however, some differences are worth mentioning. First, and somewhat unexpectedly, the weather and clothing aspects were not discussed to a large extent as a barrier or facilitator to children’s PA. This factor was addressed in a Canadian study with a sociodemographic diverse sample of child care providers [47]. It is possible that a Swedish preschool situated in a more ethnically diverse area with parents not yet used to the Swedish climate and conditions in different seasons would have addressed clothing as a greater issue. Second, gender differences for PA were not discussed in our sample, but this has been raised in previous qualitative research where boys were perceived as more active than girls [28]. In addition, in a Malawian study, although PA was seen as positive for both boys and girls, some parents thought that older girls should not be too active when growing up as this might not be culturally acceptable [48]. Thus, culture differences regarding PA in boys and girls are important to acknowledge when performing interventions in areas where children represent many cultures so that misconceptions can be avoided. Finally, the skewed gender distribution in our sample is a limitation. However, for the teachers, it is similar to the distribution in Swedish preschools (ie, only 4% of the annual employees constitute of men) [16]. The absence of males working in the preschool setting was also recognized as a gap in the literature by Hesketh et al [28]. As research has shown that fathers play an important role for children’s PA in the home environment [49], it is possible that male caregivers could have a positive impact on children’s PA in the preschool setting; however, this needs further investigation.

### Future Perspectives for an App in the Preschool Setting

There are a lot of reports showing that screen-based behaviors are becoming more prominent in younger ages, with preschoolers spending about 2 hours per day in front of a screen [8,10,50]. Therefore, the creation of an app targeting this age group is somewhat contradicting. However, an app encouraging children to be active may serve as a competitor to apps promoting sedentary behaviors in children, and it may also provide important information and support to adults’ promotion of PA in this age group. The preschool environment is an essential arena from where we can work to prevent lifestyle inequalities in young children. This is especially important as the level of childhood obesity in the preschool age group is a global concern [51]. Finding solutions to work against impaired health in young children must be a global priority. Thus, providing preschools with optimal conditions for PA is key. As changing the physical environment of already established preschools is not always feasible, an app could support teachers in the preschools to provide sufficient daily PA of different intensity regardless of physical and structural barriers. However, the development of such app features needs to be investigated further and tested in heterogeneous samples.

### Conclusions

This study identified facilitators and barriers for PA in preschoolers and provides suggestions for how an app can be used in the preschool setting to promote PA. The children spent long hours at preschool, and thus, the majority of their PA was performed there. Given opportunities, children were spontaneously active, although the amount of higher-intensity PA was perceived as low by teachers. Small indoor areas and a restricted outdoor space were identified as the main barriers for PA. Facilitators for PA were engaging teachers, high staff density, and scheduled time for planning PA. Future tailoring of interventions in the preschool setting needs to work around these barriers and support the facilitators. In such work, an app could serve as a source of inspiration for PA in different settings and thus reduce environmental and structural inequalities in the preschool setting.

## Appendix

Multimedia Appendix 1 The consolidated criteria for reporting qualitative research checklist.

Multimedia Appendix 2 Interview questions to teachers and parents.

## Abbreviations

MINISTOP: the mobile-based intervention intended to stop obesity in preschoolers
mHealth: mobile health
MVPA: moderate-to-vigorous physical activity
PA: physical activity

## References

[1] Carson V, Lee EY, Hewitt L, Jennings C, Hunter S, Kuzik N, Stearns JA, Unrau SP, Poitras VJ, Gray C, Adamo KB, Janssen I, Okely AD, Spence JC, Timmons BW, Sampson M, Tremblay MS (2017). Systematic review of the relationships between physical activity and health indicators in the early years (0-4 years). BMC Public Health.

[2] Leppänen MH, Nyström CD, Henriksson P, Pomeroy J, Ruiz JR, Ortega FB, Cadenas-Sánchez C, Löf M (2016). Physical activity intensity, sedentary behavior, body composition and physical fitness in 4-year-old children: results from the ministop trial. Int J Obes (Lond).

[3] Telama R, Yang X, Leskinen E, Kankaanpää A, Hirvensalo M, Tammelin T, Viikari JS, Raitakari OT (2014). Tracking of physical activity from early childhood through youth into adulthood. Med Sci Sports Exerc.

[4] Jones RA, Hinkley T, Okely AD, Salmon J (2013). Tracking physical activity and sedentary behavior in childhood: a systematic review. Am J Prev Med.

[5] Aubert S, Barnes JD, Abdeta C, Abi Nader P, Adeniyi AF, Aguilar-Farias N, Andrade Tenesaca DS, Bhawra J, Brazo-Sayavera J, Cardon G, Chang CK, Delisle Nyström C, Demetriou Y, Draper CE, Edwards L, Emeljanovas A, Gába A, Galaviz KI, González SA, Herrera-Cuenca M, Huang WY, Ibrahim IA, Jürimäe J, Kämppi K, Katapally TR, Katewongsa P, Katzmarzyk PT, Khan A, Korcz A, Kim YS, Lambert E, Lee EY, Löf M, Loney T, López-Taylor J, Liu Y, Makaza D, Manyanga T, Mileva B, Morrison SA, Mota J, Nyawornota VK, Ocansey R, Reilly JJ, Roman-Viñas B, Silva DA, Saonuam P, Scriven J, Seghers J, Schranz N, Skovgaard T, Smith M, Standage M, Starc G, Stratton G, Subedi N, Takken T, Tammelin T, Tanaka C, Thivel D, Tladi D, Tyler R, Uddin R, Williams A, Wong SHS, Wu C, Zembura P, Tremblay MS (2018). Global matrix 3.0 physical activity report card grades for children and youth: results and analysis from 49 countries. J Phys Act Health.

[6] Delisle Nyström C, Larsson C, Alexandrou C, Ehrenblad B, Eriksson U, Friberg M, Hagströmer M, Lindroos AK, Nyberg G, Löf M (2018). Results from Sweden's 2018 report card on physical activity for children and youth. J Phys Act Health.

[7] Chaput JP, Colley RC, Aubert S, Carson V, Janssen I, Roberts KC, Tremblay MS (2017). Proportion of preschool-aged children meeting the Canadian 24-Hour Movement Guidelines and associations with adiposity: results from the Canadian Health Measures Survey. BMC Public Health.

[8] De Craemer M, McGregor D, Androutsos O, Manios Y, Cardon G (2018). Compliance with 24-h movement behaviour guidelines among Belgian pre-school children: the ToyBox-study. Int J Environ Res Public Health.

[9] Cliff DP, McNeill J, Vella SA, Howard SJ, Santos R, Batterham M, Melhuish E, Okely AD, de Rosnay M (2017). Adherence to 24-hour movement guidelines for the early years and associations with social-cognitive development among Australian preschool children. BMC Public Health.

[10] Berglind D, Hansson L, Tynelius P, Rasmussen F (2017). Levels and patterns of objectively measured physical activity and sedentary time in 4-year-old Swedish children. J Phys Act Health.

[11] Temple M, Robinson JC (2014). A systematic review of interventions to promote physical activity in the preschool setting. J Spec Pediatr Nurs.

[12] Mehtälä MA, Sääkslahti AK, Inkinen ME, Poskiparta ME (2014). A socio-ecological approach to physical activity interventions in childcare: a systematic review. Int J Behav Nutr Phys Act.

[13] Pate RR, Brown WH, Pfeiffer KA, Howie EK, Saunders RP, Addy CL, Dowda M (2016). An intervention to increase physical activity in children: a randomized controlled trial with 4-year-olds in preschools. Am J Prev Med.

[14] Peirson L, Fitzpatrick-Lewis D, Morrison K, Ciliska D, Kenny M, Usman Ali M, Raina P (2015). Prevention of overweight and obesity in children and youth: a systematic review and meta-analysis. CMAJ Open.

[15] Peden ME, Okely AD, Eady MJ, Jones RA (2018). What is the impact of professional learning on physical activity interventions among preschool children? A systematic review. Clin Obes.

[16] The Swedish National Agency for Education.

[17] Fjeldsoe BS, Marshall AL, Miller YD (2009). Behavior change interventions delivered by mobile telephone short-message service. Am J Prev Med.

[18] Stephens J, Allen J (2013). Mobile phone interventions to increase physical activity and reduce weight: a systematic review. J Cardiovasc Nurs.

[19] Moore HJ, Hillier FC, Batterham AM, Ells LJ, Summerbell CD (2014). Technology-based dietary assessment: development of the Synchronised Nutrition and Activity Program (SNAP). J Hum Nutr Diet.

[20] Joiner KL, Nam S, Whittemore R (2017). Lifestyle interventions based on the diabetes prevention program delivered via eHealth: a systematic review and meta-analysis. Prev Med.

[21] Ludwig K, Arthur R, Sculthorpe N, Fountain H, Buchan DS (2018). Text messaging interventions for improvement in physical activity and sedentary behavior in youth: systematic review. JMIR Mhealth Uhealth.

[22] Schoeppe S, Alley S, Van Lippevelde W, Bray NA, Williams SL, Duncan MJ, Vandelanotte C (2016). Efficacy of interventions that use apps to improve diet, physical activity and sedentary behaviour: a systematic review. Int J Behav Nutr Phys Act.

[23] Downing KL, Salmon J, Hinkley T, Hnatiuk JA, Hesketh KD (2018). Feasibility and efficacy of a parent-focused, text message-delivered intervention to reduce sedentary behavior in 2- to 4-year-old children (Mini Movers): pilot randomized controlled trial. JMIR Mhealth Uhealth.

[24] Militello L, Melnyk BM, Hekler EB, Small L, Jacobson D (2016). Automated behavioral text messaging and face-to-face intervention for parents of overweight or obese preschool children: results from a pilot study. JMIR Mhealth Uhealth.

[25] Nyström CD, Sandin S, Henriksson P, Henriksson H, Trolle-Lagerros Y, Larsson C, Maddison R, Ortega FB, Pomeroy J, Ruiz JR, Silfvernagel K, Timpka T, Löf M (2017). Mobile-based intervention intended to stop obesity in preschool-aged children: the MINISTOP randomized controlled trial. Am J Clin Nutr.

[26] Rutberg S, Lindqvist AK (2018). Active school transportation is an investment in school health. Health Behav Policy Rev.

[27] Lindqvist AK, Rutberg S (2018). One step forward: development of a program promoting active school transportation. JMIR Res Protoc.

[28] Hesketh KR, Lakshman R, van Sluijs EM (2017). Barriers and facilitators to young children's physical activity and sedentary behaviour: a systematic review and synthesis of qualitative literature. Obes Rev.

[29] Tong A, Sainsbury P, Craig J (2007). Consolidated criteria for reporting qualitative research (COREQ): a 32-item checklist for interviews and focus groups. Int J Qual Health Care.

[30] Braun V, Clarke V (2006). Using thematic analysis in psychology. Qual Res Psychol.

[31] Hesketh KR, Griffin SJ, van Sluijs EM (2015). UK Preschool-aged children's physical activity levels in childcare and at home: a cross-sectional exploration. Int J Behav Nutr Phys Act.

[32] Hinkley T, Salmon J, Crawford D, Okely AD, Hesketh KD (2016). Preschool and childcare center characteristics associated with children's physical activity during care hours: an observational study. Int J Behav Nutr Phys Act.

[33] Hinkley T, Salmon J, Okely AD, Hesketh K, Crawford D (2012). Correlates of preschool children's physical activity. Am J Prev Med.

[34] Määttä S, Konttinen H, Haukkala A, Erkkola M, Roos E (2017). Preschool children's context-specific sedentary behaviours and parental socioeconomic status in Finland: a cross-sectional study. BMJ Open.

[35] Berglind D, Tynelius P (2017). Objectively measured physical activity patterns, sedentary time and parent-reported screen-time across the day in four-year-old Swedish children. BMC Public Health.

[36] McWilliams C, Ball SC, Benjamin SE, Hales D, Vaughn A, Ward DS (2009). Best-practice guidelines for physical activity at child care. Pediatrics.

[37] Bower JK, Hales DP, Tate DF, Rubin DA, Benjamin SE, Ward DS (2008). The childcare environment and children's physical activity. Am J Prev Med.

[38] Trost SG, Ward DS, Senso M (2010). Effects of child care policy and environment on physical activity. Med Sci Sports Exerc.

[39] The Swedish National Agency for Education.

[40] Niantic Inc.

[41] Lindqvist AK, Castelli D, Hallberg J, Rutberg S (2018). The praise and price of Pokémon go: a qualitative study of children's and parents' experiences. JMIR Serious Games.

[42] Rothman AJ (2004). "Is there nothing more practical than a good theory?": Why innovations and advances in health behavior change will arise if interventions are used to test and refine theory. Int J Behav Nutr Phys Act.

[43] Downing KL, Salmon J, Hinkley T, Hnatiuk JA, Hesketh KD (2017). A mobile technology intervention to reduce sedentary behaviour in 2- to 4-year-old children (Mini Movers): study protocol for a randomised controlled trial. Trials.

[44] Delisle C, Sandin S, Forsum E, Henriksson H, Trolle-Lagerros Y, Larsson C, Maddison R, Ortega FB, Ruiz JR, Silfvernagel K, Timpka T, Löf M (2015). A web- and mobile phone-based intervention to prevent obesity in 4-year-olds (MINISTOP): a population-based randomized controlled trial. BMC Public Health.

[45] Duvinage K, Ibrügger S, Kreichauf S, Wildgruber A, De Craemer M, De Decker E, Androutsos O, Lateva M, Iotova V, Socha P, Zych K, Mouratidou T, Mesana Graffe MI, Manios Y, Koletzko B, ToyBox-study group (2014). Developing the intervention material to increase physical activity levels of European preschool children: the ToyBox-study. Obes Rev.

[46] Choo EK, Garro AC, Ranney ML, Meisel ZF, Morrow Guthrie K (2015). Qualitative research in emergency care part I: research principles and common applications. Acad Emerg Med.

[47] Copeland KA, Sherman SN, Kendeigh CA, Saelens BE, Kalkwarf HJ (2009). Flip flops, dress clothes, and no coat: clothing barriers to children's physical activity in child-care centers identified from a qualitative study. Int J Behav Nutr Phys Act.

[48] Pulakka A, Ashorn P, Gondwe A, Phiri N, Ashorn U (2015). Malawian parents' perceptions of physical activity and child development: a qualitative study. Child Care Health Dev.

[49] Zahra J, Sebire SJ, Jago R (2015). "He's probably more Mr. sport than me"--a qualitative exploration of mothers' perceptions of fathers' role in their children's physical activity. BMC Pediatr.

[50] Hinkley T, Brown H, Carson V, Teychenne M (2018). Cross sectional associations of screen time and outdoor play with social skills in preschool children. PLoS One.

[51] Skinner AC, Ravanbakht SN, Skelton JA, Perrin EM, Armstrong SC (2018). Prevalence of obesity and severe obesity in us children, 1999-2016. Pediatrics.

