# Supplementary material for: Physical Activity and Mobile Phone Apps in the Preschool Age: Perceptions of Teachers and Parents
Source: JMIR Mhealth Uhealth. 2019 Apr 17;7(4):e12512. doi: 10.2196/12512 (PMC6492065; doi:10.2196/12512)
Supplement: Multimedia Appendix 2 [file mhealth_v7i4e12512_app2.pdf]

Questions to parents regarding physical activity in preschoolers and an app to promote PA

1. How would you describe physical activity (PA) in children?
2. What type of PA do you think preschoolers need?
3. How much PA do you think they need and why?
4. What do you do for your child to be physically active? (weekdays/weekend/holidays/different seasons)?
5. When and where would you say that your child is physically active?
6. When is it difficult to make time for PA in your family? How do you handle that?
7. What are your thoughts regarding screen time in this age group?
8. What facilitates PA?
9. What do you think about an app to promote PA in children?
10. What features would you want such an app to have?
11. When would you use such an app?

Now I will ask about the PA at the preschool

12. How do you perceive the activities that the preschool offers?
13. Do you think the children could be more physically active in the preschool?
14. What type of PA do you think they need more of?
15. What could the staff do to increase the PA?

Questions to teachers regarding physical activity in preschoolers and an app to promote PA

1. What would you say is PA for children 3 to 5 years old?
2. What type of PA do you think children of this age need and why?
3. How much PA do you think they need and why?
4. In what way do you think the preschool contributes to satisfy children's need for PA?
5. How do you encourage PA in the preschool (difference between the seasons)?
6. When is it difficult to make time for PA in the preschool? How do you handle that?
7. What would have to be different for you to be able to encourage PA more often?
8. What type of PA do they need more of?
9. What do you think about the PA that is offered here at the preschool?
10. If you could change one thing here to promote more PA, what would that be?
11. If you were to create an app that could support you to promote the children's PA here at the preschool, what functions and features would that app have?
12. Do you think an app could be a supportive tool to promote PA in the preschool? When?
13. And finally, what PA do you prefer the most to do with the children at the preschool?
